# Functional Disease of the Heart

**Published:** 1873-04-01

**Authors:** N. S. Davis

**Affiliations:** Professor of Clinical Medicine, Etc.


### Clinical Lectures.

### FUNCTIONAL DISEASE OF THE HEART.

LECTURE DELIVERED IN MEDICAL WARDS OF MERCY HOSPITAL. BY N. S. DAVIS, M.D., PROFESSOR OF CLINICAL MEDICINE, ETC.

Gentlemen :—The case before you is that of a young woman, who has been sent here by her attending physician, more especially for the purpose of eliciting our views in regard to the diagnosis. She presents a nervous temperament; is thin in flesh; moderately spanæmic; bowels costive; respiration quiet and easy when at rest; action of the heart steady and regular, but easily disturbed by quick exercise or excitement, and sometimes palpitating violently when she is entirely quiet, or even lying in bed. Her appetite is not good, and she says she can take but little beside bread and tea; and even these cause a sense of heaviness and discomfort in the stomach for some time after each meal. Her menstrual periods are natural; but she complains of weariness, inability to sleep well, especially in the first part of the night; and frequent paroxysms of mental apprehension and cardiac palpitation, when alone and quiet, much more than when occupied. For several years past she has been so employed as to keep her closely confined in warm rooms, and has indulged in the free use of strong tea. For several weeks past she has suffered so much from the sense of weakness, mental anxiety, and cardiac excitement that she has omitted all attempts at work, her mind being impressed with the idea that she has serious organic disease of the heart. To determine whether this idea is correct or not is the principle object of her coming before you to-day. I have given you, briefly, her present symptoms and past history, and will now note what can be learned by auscultation and percussion. Application of the stethoscope reveals nothing unnatural in the respiratory sounds in any part of the chest, unless it be a slight prolongation of the respiratory murmur in the infra-clavicular region of the left side. Neither is there a perceptible alteration in the resonance, as indicated by percussion.

On applying the ear, or stethoscope, over the cardiac region, we find the rhythm or succession of phenomena natural, but the systolic action is quick, short and sharp, and the first sound a little exaggerated, though not rough. The sound in the aorta, especially near the arch, is also louder than natural, but is neither rough nor harsh.

The essential symptoms of this case then may be stated as follows : general feeling of weakness, with mental depression and anxiety; indigestion; moderate constipation; nervous excitability and cardiac palpitations, more especially when quiet and alone; a sense of fulness or oppression in the upper part of the chest, and occasional sharp pains under the left breast; insomnia during the first half of the night, and a slight exaggeration of the cardiac sounds. Do these phenomena indicate any form of disease of the heart? Valvular disease in any of the cardiac openings always causes more or less roughness, and, generally, irregularity in rythm, and uniformly increases the symptoms when the patient attempts exercise, and diminishes them when at rest. Simple hypertrophy, without cardiac disease, causes constant increase, both in the force and direction of the systolic action of the heart, as well as increased shortness of breath on attempting exercise. If the muscular structure of the heart had undergone degeneration, fatty or otherwise, the effect would be to render its action weak, often irregular, and always tremulous or disturbed on taking exercise. You thus observe one symptom common to all organic diseases of the heart, namely, increased trouble, both in respiration and the abnormal action of the heart by exercise. On the contrary, nervous, sympathetic and functional disturbances of the heart are more apt to trouble the patient when quiet and alone, or after eating, than while the mind and body are both occupied with moderate exercise. Apply these rules to the patient before you, and you will have little difficulty in arriving at a satisfactory diagnosis. The quick, systolic action of her heart, the cardiac fluttering and mental depression when alone, and sitting or lying still, the temporary excitement after eating, and the various morbid feelings in the upper part of the chest, as well as in the cardiac region, that prevent sleep in the first part of the night, all show that the cardiac disturbance is purely functional, arising from a morbid sensitiveness of the cardiac and cœliac plexuses of the sympathetic and the pneumo-gastric nerves. The morbid condition of these portions of the nervous system, and the consequent impairment of digestion and nutrition, in this case, has resulted from the co-operation of two causes, namely : close, long continued confinement within doors, and the too liberal use of tea. The latter is one of the most direct and pure excitants of the nervous structures that we possess; and when used persistently and freely, especially by those who take but little exercise in the open air, it often induces that morbid susceptibility of the nervous centers, which causes loss of appetite for nutritious food, insomnia, tremulousness, palpitations, pain under the left breast, etc. This young woman says that I examined her about two years since, on account of symptoms similar to those of which she now complains, and that I then assured her that she had no organic disease of the heart. I must repeat the same assurance to her to-day, with the addition that she can be relieved of all her annoying symptoms, and regain fair health, if she will permanently correct the faults in her mode of life. If she will take from one to two hours of active exercise in the open air daily, occupy a sleeping room sufficiently capacious and well ventilated, take nutritious and digestible food regularly at the meal-times, and entirely avoid the use of tea, coffee, and all other nervous excitants and exhilarants, in six weeks she will be able to sleep as soundly, and feel as free from apprehensions of "heart disease," as any of us. She may be aided in attaining so desirable a result by a judicious use of medicine; but the latter alone can never afford permanent relief, while the causes that have been most influential in producing disease, are permitted to continue operative. One of the following pills, taken just before each meal-time, would improve the tone and efficiency of the digestive organs, and promote regularity in the intestinal evacuations :

℞.—Ext. hyosciamus, 40 grs.
Ext. scutellaria, 40 grs.
Sulph. ferri, 40 grs.
Pulv. aloes, 15 grs.
Blue mass, 10 grs.
Mix. Divide into 40 pills.

And two teaspoonfuls of the following solution would ensure much earlier and better sleep at night:

℞.—Bromide pot., ʒ iv.

Tinct. digitalis, ʒ iv.

Syrup prunus virg., ℥ ii.

Aquæ ℥ iss.

